# Submuscular and Pre-pectoral ADM Assisted Immediate Breast Reconstruction: A Literature Review

**DOI:** 10.3390/medicina56060256

**Published:** 2020-05-26

**Authors:** Roberto Cuomo

**Affiliations:** Santa Maria Alle Scotte Hospital, Plastic and Reconstructive Surgery Unit, Department of Medicine, Surgery and Neuroscience, University of Siena, Mario Bracci Street, 53100 Siena, Italy; robertocuomo@outlook.com

**Keywords:** acellular dermal matrix, ADM, breast reconstruction, pre-pectoral, submuscular

## Abstract

*Background and objectives*: Breast cancer treatment has deeply changed in the last fifty years. Acellular dermal matrices (ADMs) were introduced for breast reconstruction, with encouraging results, but with conflicting reports too. The present paper aims to summarize the current data on breast reconstruction using acellular dermal matrices. *Materials and Methods*: We reviewed the literature regarding the use of ADM-assisted implant-based breast reconstruction. *Results*: The main techniques were analyzed and described. *Conclusions*: Several authors have recently reported positive results. Nevertheless, an increased complications’ rate has been reported by other authors. Higher cost compared to not-ADM-assisted breast reconstruction is another concern.

## 1. Introduction

The use of acellular dermal matrix (ADM) for breast reconstruction was described by Salzberg in 2006 [1] and by Dieterich in 2015 [2,3]. Acellular dermal matrices (ADMs) are made from fetal bovine, porcine or human cadaver and represent a sort of scaffold that autologous cells can colonize [4,5].

Immediate breast reconstruction (IBR) received an important boost in popularity as a consequence of the advent of ADMs [2,6,7,8,9,10,11,12,13,14,15,16,17,18,19]. The use of ADMs showed encouraging results but conflicting reports as well [20,21,22,23,24,25,26,27,28,29,30,31,32,33,34,35,36,37,38,39,40,41,42]. ADMs-assisted breast reconstruction can be divided into pre-pectoral and submuscular. The present narrative review summarizes the current evidences on immediate breast reconstruction using ADM.

## 2. Materials and Methods

We performed a review of literature, starting from 2006, by searching on PubMed “acellular dermal matrix” and “breast reconstruction”, focusing on surgical techniques, outcomes and complications’ rate, in order to better understand the evidences on this topic.

## 3. Results

### 3.1. Acellular Dermal Matrix (ADM) and Breast Reconstruction

Immediate breast reconstruction (IBR) has radically changed the concept of breast cancer to the extent that a patient admitted to surgery for breast cancer is discharged without the impact of breast amputation.

The main advantages of IBR can be summarized as lower costs for the healthcare system (shorter healing time and fewer hospitalizations) and the elimination of tissue expansion time [43,44,45,46,47]. Despite this, several studies have reported high rates of complications linked to immediate breast reconstruction [2,7,48,49,50,51]. Many authors analyzed these aspects, underlining the safety of IBR and the good outcomes reached with careful patient selection and adherence to surgical techniques [2,52,53,54,55,56,57,58,59,60,61,62,63]. IBR has similar postoperative complications to delayed breast reconstructions with tissue expander and implant, although tissue expander/submuscular implant has been the most popular reconstruction strategy [43,44,64,65,66,67].

The American Society of Plastic Surgeons reported the use of ADMs in about 50% of breast reconstruction in 2012 [68], and these data were confirmed over time [69].

Recent research confirmed good outcomes for ADMs assisted IBR as underlined by Negeborn et al. [35,70] and Carminati et al. [21], with acceptable risks of infection. This risk is higher in obese patients [21]. Improved aesthetic outcomes following ADM use in tissue expander/implant-based breast reconstruction was assessed by Ibrahim et al. [71]. ADM may improve breast volume, placement and inframammary fold definition [72].

The main disadvantage of this kind of procedure is the high costs, as shown by Gravina et al. [24]. They analyzed the different characteristics of the main ADMs and their alternatives, underlining the good aesthetic outcomes and the benefits of single-stage procedures, but these aspects are balanced with high costs and an increased risk of infection and overall surgical complication [24].

Many authors agree that IBR received an important boost in popularity as a consequence of the advent of AMDs [2,6,7,8,9,10,11,12,13,14,15,16,17,18]. ADM-assisted breast reconstruction can be divided into submuscular and pre-pectoral.

### 3.2. Submuscular ADM-Assisted Breast Reconstruction

In submuscular breast reconstruction, the surgeon can place an ADM to cover the inferior pole of the implant [73,74,75,76]. This is helpful in the following situations:

(1) The breast has a good volume, and the surgeon needs to use an implant of adequate volume for immediate reconstruction, but the inferior pole of the implant cannot be completely covered by the Pectoralis Major [9,10,57,77,78].

(2) To prevent the need of major elevation of muscle, reducing postoperative pain [77,79,80,81].

Partial muscle coverage is important to obtain a more natural shape, releasing the constriction of the inferior aspect of pectoralis muscle but less coverage of prostheses in the lateral-inferior aspect can occur in some cases [77,82].

Lateral control of the implant position can be obtained by using Serratus or minimizing the lateral dissection during the mastectomy, but this may not be enough. In these cases, the use of an ADM allows surgeons to better control the stability of the breast implant both in immediate and delayed breast reconstruction [77,83,84,85,86].

**Figure 1:**
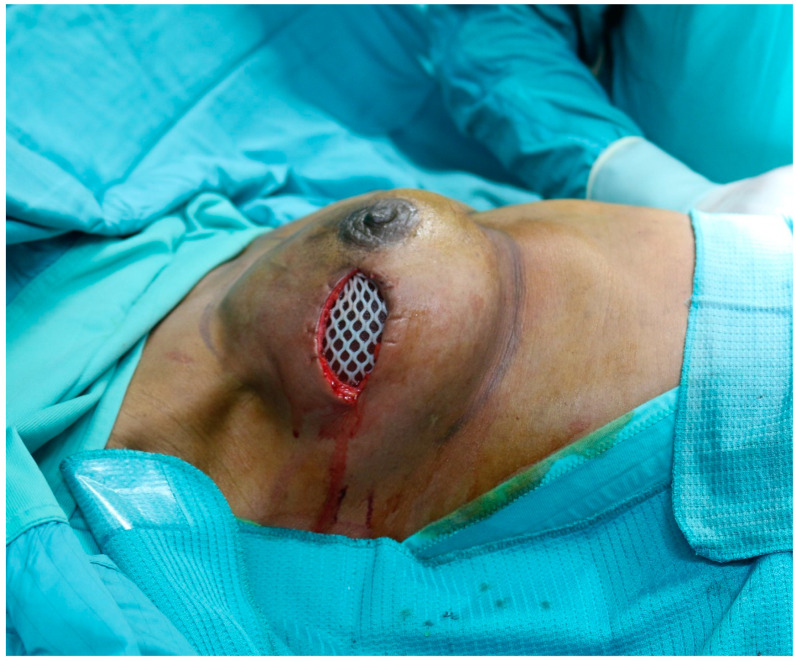
Meshed ADM used to cover the inferolateral aspect of the implant in submuscular breast reconstruction. ADM: Acellular dermal matrix.

The submuscular breast reconstruction performed using ADM to cover the lateral or the inferior pole of neo-breast is routinely referred to as dual-plane reconstruction (see Figure 1). The most common anti-aesthetic reports is the muscle retraction deformity; this can be avoided by suturing the ADM at the inferior border of the muscle, from the four to eight o’clock position [77,84,87].

Lateral sutures can be used between the skin flap and the chest wall to better close the dead space and improve the lateral contour, but the skin thickness should be carefully evaluated, in order to avoid quilting sutures [8,64,88,89,90,91,92].

Many authors agree that this kind of reconstruction has excellent long-term cosmetic results; the main unexpected event is the distortion or the movement of the implant with flexion of the muscle. Compared to pre-pectoral reconstruction, it is less expensive and can lead to better coverage of the upper pole of the breast. Nevertheless, it is burdened by the risk of upper migration of the implant and more pain due to muscle detachment [2,7,77,83,89,90,91].

### 3.3. Pre-Pectoral ADM-Assisted Breast Reconstruction

**Figure 2:**
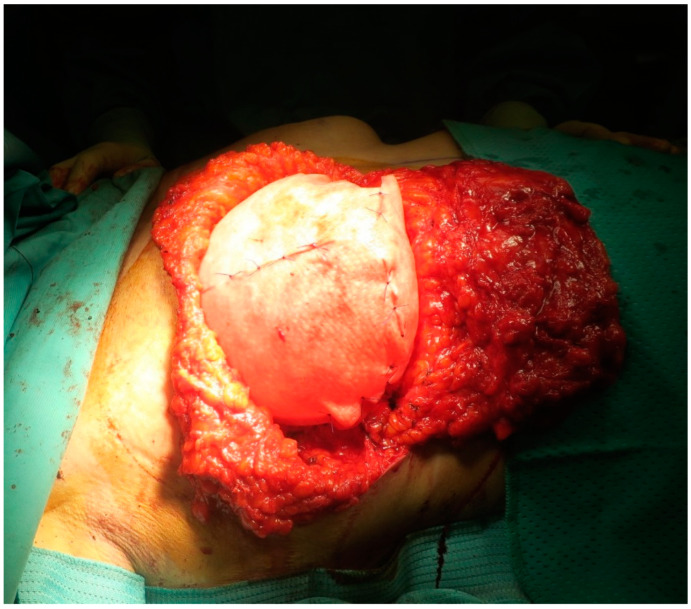
ADM-assisted pre-pectoral breast reconstruction with vertical scar.

The concept of pre-pectoral breast reconstruction (see Figure 2) can be considered as the “evolution” of breast reconstruction in terms of “tissue sparing”: As nipple-skin sparing mastectomy for the oncologic surgery, pre-pectoral breast reconstruction focuses on sparing the Pectoralis Major Muscle. ADM has a key role in this kind of procedure because it wraps (at least in the front) the implant for a complete integration in the host [93,94].

Pre-pectoral breast reconstruction was suggested in those cases where implants less than 500 cc were requested [95]. Actually, this indication has been modified, and some authors describe pre-pectoral breast reconstruction with implants over 600 cc [77].

Many authors choose the pre-pectoral breast reconstruction because the submuscular placement of the implant can lead to a result described as “contrived breast” [82,91,95,96]. This aspect is relevant and linked to a loss of muscle function; many authors, in fact, underline that patients, in particular after tissue expansion, need physiotherapy. The muscle-spearing breast reconstruction was proposed by many authors over time.

In 2013, Cheng proposed the treatment of capsular contracture using an ADM; he did not perform pre-pectoral reconstruction, but removed the contracted capsule and put ADM to cover the anterior aspect of the implant on 16 breasts. He reported only one infection by coagulase negative Staphylococcus and Mycobacterium fortuitum [97]. The reduction of incidence in capsular contracture using ADMs was underlined in time by Lardi et al., in 2017 [30], and confirmed by Liu et al., with a meta-analysis in 2020 [33].

Becker et al. (2015) reported the experience on 62 breasts covering the anterior aspect of saline implant with an ADM sutured to the muscle. The complications reported were three flap necrosis, one seroma, one infection, one hematoma and two capsular contractures [98].

In 2017, Berna firstly proposed a complete ADM coverage of the implant [93]; the implant stability was guaranteed by suturing the implant and its “envelope” to the muscle. On 100 reconstructions with this procedure, Vidya et al. underlined two hematoma, three dehiscence, one necrosis, five seromas and two implant losses [95].

The main purpose of pre-pectoral reconstruction is to save the function of Pectoralis Major, decreasing the postoperative pain and reducing the follow-up time. Other advantages are represented by minor risk in the upper migration of the implant and a better breast projection [99,100].

The main disadvantages are the high costs of these devices (which are to be added to the cost of breast implants) and the higher risk of symmastia, the rippling and an irregularity of the highest limit of the upper pole of the breast and the high risk of seroma. Several authors suggest not removing the drains until finding a maximum of 30cc for three consecutive days [18,77,101].

The dimpling of the upper pole of the breast occurs due to the thinning of the subcutaneous tissue and can be avoided with lipofilling [102] or leaving 1 cm of subcutaneous fat in selected cases [103] or harvesting tissue from the muscle [104].

### 3.4. Complications and Outcomes

Tasoulis et al. observed that ADM-assisted breast reconstruction reduces the complications’ rate [105]. Onesti et al. observed that the use of ADM reduces the inflammatory response, along with the likelihood of capsular contracture [36].

On the other hand, Lohmander et al. [106] observed that immediate IBR with ADM carried a risk of implant loss equal to conventional IBR without ADM, but was associated with more adverse outcomes, requiring surgical intervention, through an open-label, multicenter, randomized, controlled trial on 135 women. Antony et al. [107]. observed that acellular human dermis is useful in immediate tissue expander reconstruction but can lead to an increased risk of complications (seroma and reconstructive failure).

The literature data show that the complications’ rate is similar for subcutaneous and submuscular reconstruction ADM assisted, without statistical significance for major adverse events (explantation, wide infections, Baker grade III or IV contracture, and complete nipple–areola complex necrosis) [22]. Overall, the most described complications for ADMs-assisted reconstruction are seroma (up to 9% of cases), explantation (up to 6.5%) and partial nipple–areola complex (NAC) necrosis (up to 5.3%) [2,37,65,83,108,109,110,111,112].

In 2017, Kim and Bang linked the use of ADM and the mastectomy flap necrosis [28]. Powell-Brett and Goh [113] reported 10.4% cases of skin necrosis in a study with ADM-assisted immediate breast reconstruction.

This last complication should be interpreted as follows: It can occur (in some cases) for tissue ischemia during the cancer removing and the implant. Intraoperative tools to evaluate NAC viability can lower this complication’s rate, but these devices are expensive, time-consuming and not available in all centers [41,114,115,116].

The pre-pectoral breast reconstruction is burdened by the following patient complaints: rippling (up to 4.5%) and visible implants (4.3%). The submuscular breast reconstruction is burdened by postoperative pain with significant impact on daily activities (5%), implant deformity and less-natural cosmetic outcomes (until 7%) [6,93,108,117,118,119,120,121,122,123,124]. Onesti et al. suggested a modified technique in obesity patients with large breasts, using a dermal flap to cover the ADM-implant in the pre-pectoral plane, in order to improve the outcomes. Obesity and smoking are always linked to a higher risk of complications [125,126,127].

## 4. Conclusions

Pre-pectoral and submuscular breast reconstruction with the use of ADMs have no significant difference in complication rate. Particular care must be taken for seroma formation. Obesity and smoking are linked to higher risks of complication. The cost/benefit ratio should be carefully reviewed.
